# Intravenous Administration Is an Effective and Safe Route for Cancer Gene Therapy Using the *Bifidobacterium*-Mediated Recombinant HSV-1 Thymidine Kinase and Ganciclovir

**DOI:** 10.3390/ijms17060891

**Published:** 2016-06-06

**Authors:** Huicong Zhou, Zhiliang He, Changdong Wang, Tingting Xie, Lin Liu, Chuanyang Liu, Fangzhou Song, Yongping Ma

**Affiliations:** Molecular Medicine & Cancer Research Center, Department of Biochemistry & Molecular Biology, Chongqing Medical University, Yuzhong District, Yi XueYuan Road, Number 1, Chongqing 400016, China; zhczb@163.com (H.Z.); jk0072007@163.com (Z.H.); cdwhust@163.com (C.W.); 18716461018@163.com (T.X.); 15086785450@163.com (L.L.); 13883688451@163.com (C.L.); fzsongcq@163.com (F.S.)

**Keywords:** cancer, gene therapy, *Bifidobacterium*, safety, thymidine kinase, ganciclovir, vascular endothelial growth factor (VEGF)

## Abstract

The herpes simplex virus thymidine kinase/ganciclovir (HSV TK/GCV) system is one of the best studied cancer suicide gene therapy systems. Our previous study showed that caspase 3 expression was upregulated and bladder tumor growth was significantly reduced in rats treated with a combination of *Bifidobacterium* (BF) and HSV TK/GCV (BF-rTK/GCV). However, it was raised whether the BF-mediated recombinant thymidine kinase combined with ganciclovir (BF-rTK/GCV) was safe to administer via venous for cancer gene therapy. To answer this question, the antitumor effects of BF-rTK/GCV were mainly evaluated in a xenograft nude mouse model bearing MKN-45 gastric tumor cells. The immune response, including analysis of cytokine profiles, was analyzed to evaluate the safety of intramuscular and intravenous injection of BF-rTK in BALB/c mice. The results suggested that gastric tumor growth was significantly inhibited *in vivo* by BF-rTK/GCV. However, the BF-rTK/GCV had no effect on mouse body weight, indicating that the treatment was safe for the host. The results of cytokine profile analysis indicated that intravenous injection of a low dose of BF-rTK resulted in a weaker cytokine response than that obtained with intramuscular injection. Furthermore, immunohistochemical analysis showed that intravenous administration did not affect the expression of immune-associated TLR2 and TLR4. Finally, the BF-rTK/GCV inhibited vascular endothelial growth factor (VEGF) expression in mouse model, which is helpful for inhibiting of tumor angiogenesis. That meant intravenous administration of BF-rTK/GCV was an effective and safe way for cancer gene therapy.

## 1. Introduction

Gastric cancer is the fifth most common cancer, with the highest morbidity and mortality rate in the world [1]. Although surgery remains the best treatment option for potential cure only for selected patients suitable for surgery [2], the exploration of novel non-surgical treatments, including gene therapy, provides hope for the treatment of gastric cancer [2,3]. Cancer gene therapy is an important and promising area of cancer research. Generally, the vectors used in this therapy are classified into viral, nonviral, and bacterial vectors [4,5]. Viral vectors are efficient delivery agents, but toxicity and immunogenicity limit their clinical usage [6,7]. Thus, due to the safety concerns associated with viral vectors, nonviral vectors are preferred. Nonviral vectors (or synthetic vectors) are typically composed of cationic lipids or polymers, which can complex with negatively charged nucleic acids to form particles with an approximate diameter of 100 nm. This complex protects nucleic acids from degradation by nucleases [6]. Bacterial vectors have the unique potential to overcome the limitations of viral vectors by specifically targeting tumors, either external to (non-invasive species) or within tumor cells (pathogens) [8,9,10]. Despite significant progress in reconstructing vectors [2,10,11,12,13], several hurdles still prevent success in the clinic, including non-specific expression, low-efficiency delivery, and biosafety [6,14,15,16,17]; however, the safety of gene carriers remains the biggest obstacle for tumor gene therapy [6,18]. Indeed, the most urgent task in this field is to design a safe gene delivery vehicle, rather than to select a powerful target gene.

*Bifidobacterium* (BF) is an anaerobic probiotic with a variety of physiological functions, including roles in immunomodulation, prevention of cancer and infection, and nutrition [19,20]. Therefore, BF is used in the health care and food industries as a probiotic. BF can target the hypoxic environment of solid tumors and has been considered as an alternative strategy in tumor therapy [8,10,21,22,23].

A primary feature of solid cancer is internal hypoxia, where oxygen concentrations are a third of those in healthy tissues and the partial pressure of oxygen is almost zero within the capillaries of tumors with radii ranging from 150 to 200 mm [24]. Therefore, BF is an optimal cancer gene therapy vector due to the anaerobic properties. In recent years, BF has been widely used as a cancer-targeting gene therapy vector [10,21,22,25,26,27,28,29].

The herpes simplex virus thymidine kinase/ganciclovir (HSV TK/GCV) system is currently one of the best studied cancer suicide gene therapy systems [30]. The TK expressed specifically in tumor tissues can convert the non-toxic precursor GCV into the GCV-3-phosphate, a toxic substance that kills tumor cells [10]. We previously found that caspase 3 expression was upregulated and bladder tumor growth was significantly reduced in rats treated with a combination of BF and HSV TK/GCV (BF-rTK/GCV) for 15 days [10]. However, it was always questioned whether it is safe to administer live BF-rTK into the blood for cancer gene therapy.

A lesson learned from a death case of gene therapy suggested vector-induced activation of innate immunity was the major cause, leading to an acute release of inflammatory mediator (high serum levels of IL-6 and IL-10 but normal TNFα) [31,32]. The toxicity assay of gene therapeutic viral or bacterial vectors was needed for further study of the immune response to these vectors in the host [32].

However, little is currently known about the safety of BF-rTK vector with regard to the immune response. The immoderate immunogenicity was a major toxicity of gene therapy delivery vectors [6,7]. Therefore, the safety of the BF-rTK was assessed by analysis of the immune-associated cytokine profiles in BALB/c mice. Moreover, a gastric cancer model was established in the nude mouse model to evaluate the efficacy of gene therapy using the BF-rTK/GCV system. The universality of the antitumor mechanism of BF-rTK/GCV was confirmed in human an intestinal cancer colo320 xenograft model using quantitative real-time PCR (qPCR).

## 2. Results

### 2.1. pBEX-tk Is Expressed in Bifidobacterium (BF)

**Figure 1:**
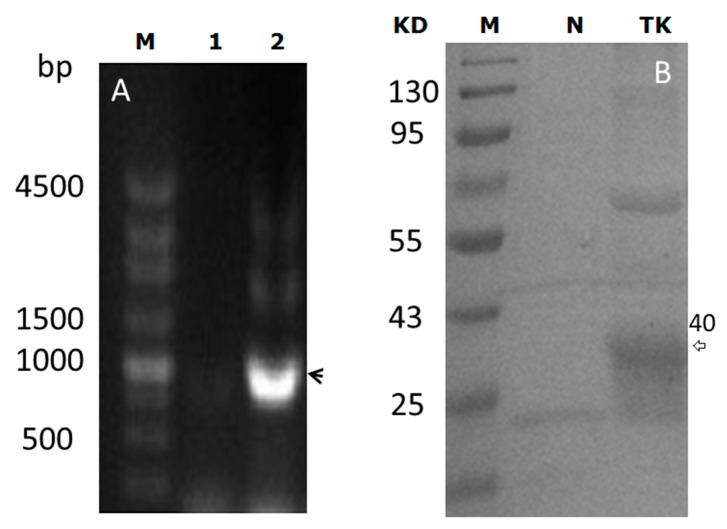
pBEX-tk was expressed in *Bifidobacterium* (BF). (**A**) PCR results. M: DNA standard molecule; lane 1: Negative control with pBEX template; lane 2: PCR product of *tk* with pBEX-tk template (black arrow); (**B**) polyacrylamide gel electrophoresis (PAGE) results. M: Protein standard molecule; lane N: Negative control of pBEX; lane TK: Purified TK expressed in pBEX-tk with His-tag (white arrow).

In order to validate the construction of the pBEX-tk plasmid, PCR and polyacrylamide gel electrophoresis (PAGE) analysis were used. PCR analysis indicated that the target gene, *tk*, was inserted into pBEX. PAGE assay confirmed that TK could be expressed successfully by BF as a 40 KD protein (Figure 1).

### 2.2. Administration of BF-rTK/GCV via Intravenous (IV) Only Minimally Induces Cytokine Expression

It has been reported that cytokine induction by gene therapy vectors is seen as a serious side effect in the patients [6,16,31,32,33,34]. Therefore, the cytokine profile assay is an important index to evaluate the safety of BF-rTK administration. The safety of BF-rTK/GCV was focused on the exiting of live bifidobacteria in blood. Therefore, the experiments were performed between only phosphate buffer saline (PBS) and BF-rTK without considering the combination of GCV in this section.

**Figure 2:**
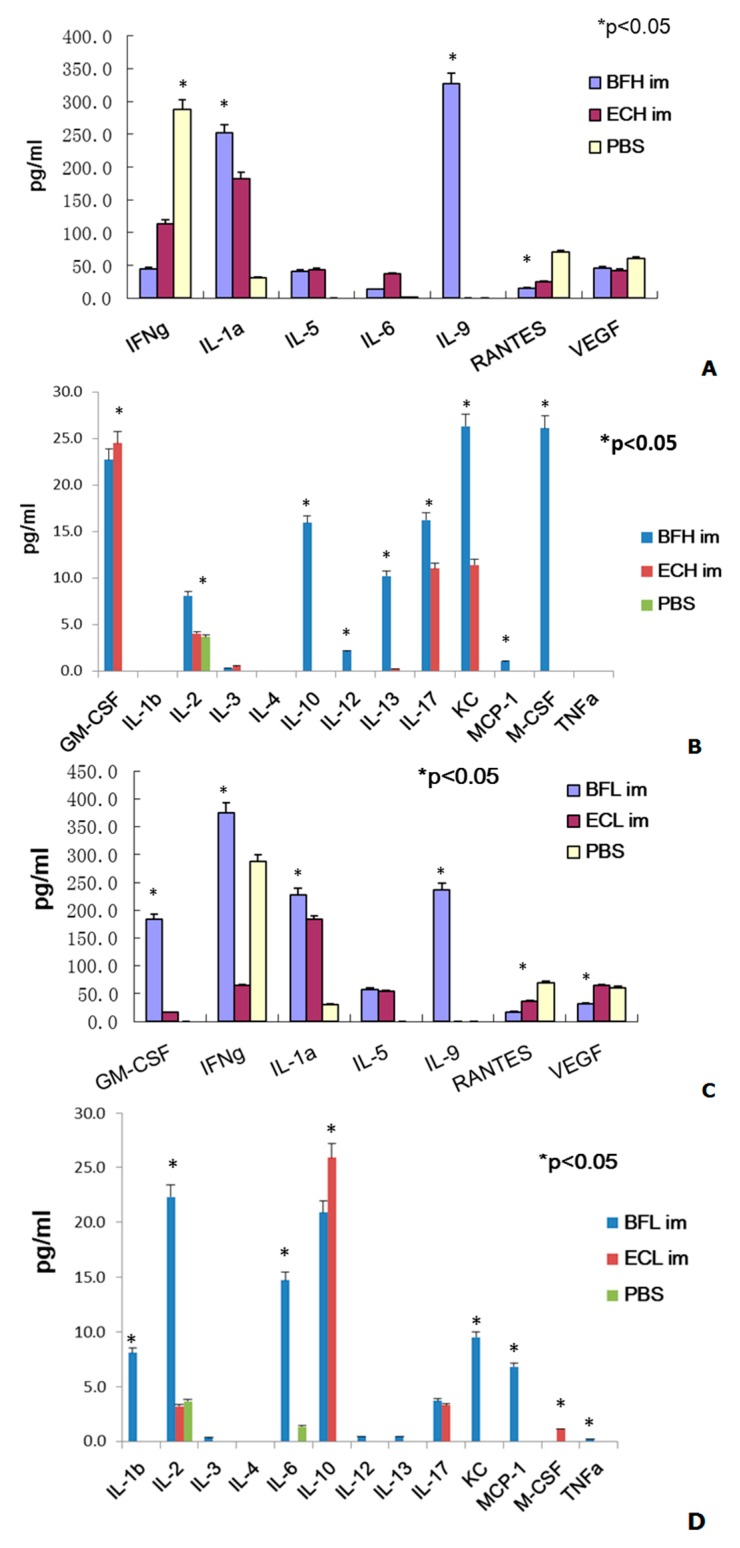
Comparing the cytokine profiles differentiation between BF-rTK and *E. coli* in BALB/c mice via intramuscular (IM) (*n* = 6). (**A**) Cytokine levels expressed in IM group with high dose of bacteria (1.0 × 10^6^ cells/mL); (**B**) Cytokine levels expressed in IM group with high dose (1.0 × 10^6^ cells/mL); (**C**) Cytokine levels expressed in IM group with low dose of bacteria (1.0 × 10^4^ cells/mL); (**D**) Cytokine levels expressed in IM group with low dose of bacteria (1.0 × 10^4^ cells/mL). BFH: BF-rTK with high dose (1.0 × 10^6^ cells/mL); BFL: BF-rTK with low dose (1.0 × 10^4^ cells/mL); ECH: *E. coli* with high dose (1.0 × 10^6^ cells/mL); ECL: *E. coli* with low dose (1.0 × 10^4^ cells/mL); im: intramuscular injection (* *p* < 0.05). PBS: phosphate buffer saline.

At first, we examined the cytokine profiles treated with different dose of BF-rTK and *E. coli* via intramuscular (IM), respectively. The cytokine profile assay indicated that the expression of nine cytokines (IL-5, IL-6, IL-9, IL-10, IL-13, IL-17, KC, GM-CSF, and M-CSF) was significantly enhanced more than 10-fold and that of three cytokines (IL-1α, IL-2, and IL-12) was only enhanced more than 2.0-fold following IM injection of the high dose of BF-rTK (1.0 × 10^6^ cells/mL) compared with the PBS control cohort (Figure 2). However, the expression of two cytokines (RANTES and VEGF) was reduced more than 5.0-fold (RANTES) and 1.3-fold (VEGF) in this group (Figure 2A). Anyway, IL-1β, IL-4 and TNFα were no detectable expression (Figure 2).

In contrast, the expression of six cytokines (IL-5, IL-6, IL-9, IL-10, KC, and GM-CSF) was upregulated more than 10-fold and that of five cytokines (IL-1α, IL-1β, IL-2, IL-17 and MCP-1) was only enhanced more than 2.0-fold following IM injection with the low dose of BF-rTK (1.0 × 10^4^ cells/mL; Figure 2). The RANTES and vascular endothelial growth factor (VEGF) expression was reduced more than 2.0-fold (Figure 2C). Only IL-4 and M-CSF were no detectable expression by IM injection of low dose of BF-rTK (Figure 2D). Obviously, TNFα was downregulated by IM injection of high dose of BF-rTK and normal in group of low dose of BF-rTK (0.2 pg/mL) (Figure 2B,D).

**Figure 3:**
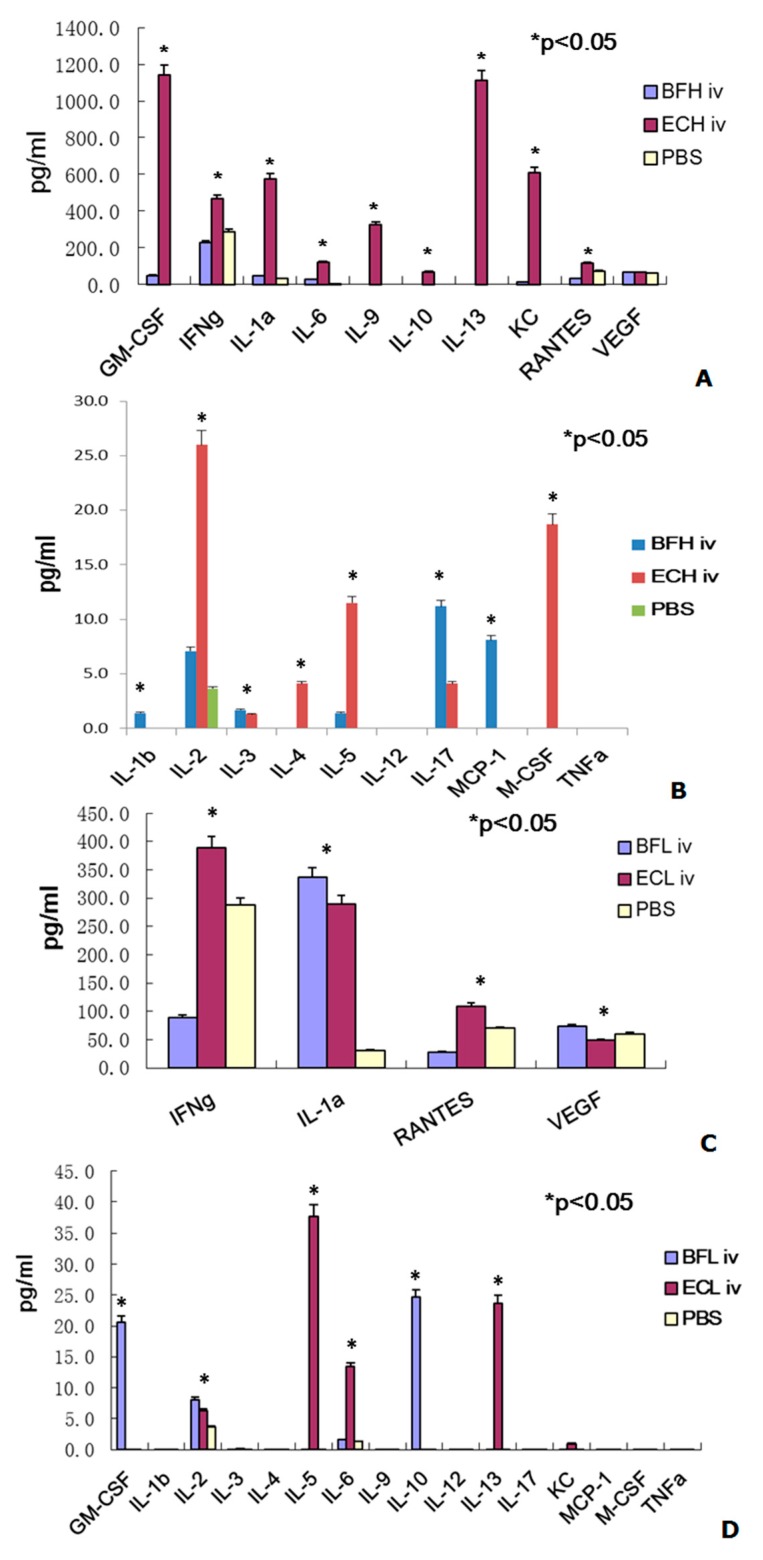
Comparing the cytokine profiles differentiation between BF-rTK and *E. coli* in BALB/c mice via intravenous (IV) (*n* = 6). (**A**) Cytokine levels expressed in IV group with high dose of bacteria (1.0 × 10^6^ cells/mL); (**B**) Cytokine levels expressed in IV group with high dose of bacteria (1.0 × 10^6^ cells/mL); (**C**) Cytokine levels expressed in IV group with low dose of bacteria (1.0 × 10^4^ cells/mL); (**D**) Cytokine levels expressed in IV group with low dose of bacteria (1.0 × 10^4^ cells/mL). BFH: BF-rTK with high dose (1.0 × 10^6^ cells/mL); BFL: BF-rTK with low dose (1.0 × 10^4^ cells/mL); ECH: *E. coli* with high dose (1.0 × 10^6^ cells/mL); ECL: *E. coli* with low dose (1.0 × 10^4^ cells/mL); iv: vein injection (* *p* < 0.05).

Similarly, four cytokines (IL-6, IL-17, KC, and GM-CSF) were upregulated more than 10-fold and two cytokines (IL-2 and MCP-1) were enhanced more than 2.0-fold by intravenous (IV) injection of the high dose of BF-rTK compared with the PBS control group (Figure 3A,B). However, the expression of RANTES was reduced more than 2-fold in this group (Figure 3A). Anyway, seven cytokines (IL-4, IL-9, IL-10, IL-12, IL-13, M-CSF and TNFα) were no detectable expression (Figure 3C,D).

In contrast, only three cytokines (IL-1α, IL-10, and GM-CSF) were upregulated more than 10-fold and only IL-2 was enhanced 2-fold by IV administration of the low dose of BF-rTK (Figure 3C,D). However, three cytokines (IL-6, IFN-γ, and RANTES) were reduced more than 2.0-fold (Figure 3A). The other twelve cytokines (IL-1α, IL-3, IL-4, IL-5, IL-9, IL-12, IL-13, IL-17, M-CSF, MCP-1, and TNFα) were no detectable expression by IV administration of low dose of BF-rTK compared with the PBS control cohort (Figure 3). However, VEGF was upregulated 1.2-fold than that of in PBS group (Figure 3A).

In contrast, five cytokines (IL-5, IL-6, IL-17, GM-CSF, and KC) in high dose of IM *E. coli* DH5α and three cytokines (IL-5, IL-10, and GM-CSF) in low dose of IM *E. coli* were upregulated more than 10-fold, respectively, compared with the PBS control (Figure 2). Two cytokines (IFN-γ and RANTES) were downregulated more than 2.0-fold in both doses of *E. coli* treatment. However, IL-1β in high *E. coli* dose and IL-17 in low *E. coli* dose were only increased more than 2.0-fold by IM administration compared with the PBS controls (Figure 2). However, nine cytokines (IL-1β, IL-5, IL-6, IL-9, IL-10, IL-13, KC, GM-CSF, and M-CSF) and four cytokines (IL-1β, IL-5, IL-6, and IL-13) were upregulated more than 10-fold in the high and low doses IV *E. coli* cohort compared with the PBS controls. Three cytokines (IL-2, IL-4, and IL-17) in high dose of IV BF-rTK and IL-2 in low dose of IV *E. coli* were only upregulated more than 2.0-fold compared with the PBS group (Figure 3).

When the cytokine ratio of BF-rTK/*E. coli* DH5α were analyzed, we found that four cytokines (IL-9, IL-10, IL-13, and M-CSF) were expressed more than 10-fold and three cytokines (IL-2, IL-12, and KC) were expressed more than 2.0-fold by IM administration of the high dose of BF-rTK group (Figure 2A,B). Whereas, four cytokines (IL-6, IL-9, KC, and GM-CSF) were expressed more than 10-fold and three cytokines (IL-2, IFN-γ, and MCP-1) were expressed more than 2.0-fold by IM administration of the low dose of BF-rTK group compared with the same dose of *E. coli* DH5α treatment via IM (Figure 2C,D).

Similarly, the ratio of IL-17 was more than 2.8-fold by IV administration of the high BF-rTK dose compared with the same dose of *E. coli* DH5α treatment (1.0 × 10^6^ cells/mL; Figure 2B) and two cytokines (IL-10 and GM-CSF) were upregulated more than 21-fold compared with the low *E. coli* dose (1.0 × 10^4^ cells/mL; Figure 3D). As a summary, the expression of IL-6 and IFN-γ (high dose of BF-rTK) and that of RANTES and VEGF (low dose of BF-rTK) were reduced more than 2.5-fold and 2.0-fold, respectively, compared with the same dose of *E. coli* DH5α treatment via IV. What’s surprising was that the ratio of IL-9, KC, IL-13, and GM-CSF were 323-, 610-, 1112-, and 1140-fold when *E. coli* DH5α was administrated via IV with high dose compared to that of BF-rTK group. The results indicated that IV administration of high dose of *E. coli* vigorously evoked both cytokine response as well as an inflammatory response, possibly because it contains LPS (lipopolysaccharide, an endotoxin). Thus, BF, a gram-positive organism, was safer than a gram-negative organism for IV gene therapy for solid tumors.

When we compared the cytokine ratio of BF-rTK/*E. coli* DH5α, the results indicated that only GM-CSF and IL-10 were significantly upregulated more than 20-fold following IV injection of the low dose of BF-rTK (Figure 3). However, IL-9 expression was upregulated more than 236-fold via IM route with both doses (Figure 2). It implied that the lower dose of BF-rTK by IV route resulted in upregulation of only two cytokines (GM-CSF and IL-10) less than 20-fold. It was much better compared to other dose/route combinations. This confirmed our conclusion that IV administration of the low dose of BF-rTK was the safest cancer gene therapy option.

The trends in the cytokine profiles were easy to identify if the data for the concentration and injection route effects were combined. Therefore, the results indicate that IV administration of BF-rTK at lower doses could minimize the tumor gene therapy cytokine response.

Taken together, the BF-rTK/GCV system is a better target gene carrier for gene therapy targeting solid tumors. The cytokine profile data suggested that it is most beneficial for reducing the host immune response to BF-rTK when injected IV at a low dose. Moreover, if the BF-rTK survived in the tumor via IV administration, it would be assumed that BF-rTK stimulated the production of higher concentrations of cytokines (e.g., IL-1α, IL-1β, IL-2, IL-10, IL-17, GM-CSF, and MCP-1). These cytokines are beneficial for evoking specific immune responses and enhancing antitumor effects, including the recruitment of immune cells to tumor tissues. Furthermore, the specific immune responses occurred only in tumor tissue around the live BF-rTK. Additionally, the downregulated expression of VEGF would inhibit tumor angiogenesis. Thus, IV injection of the low dose of BF-rTK is a safe method of administration for solid tumor gene targeting therapy.

### 2.3. Intramuscular (IM) Injection of a High Dose of BF-rTK Induces TLR2 Expression

Innate recognition of bacteria is a key step in the activation of inflammation. Toll-like receptor (TLR) plays critical roles in both the immune response and in apoptosis activation. TLR2 and TLR4 detect a range of bacterial components and harmonize the inflammatory responses through nuclear factor-kappa B (NF-κB). Previous research has showed that enterobacteria-activated leukocytes and endothelial cells in a TLR4/MD-2-dependent manner via LPS [35]. Gram-positive bacteria activated cells only at high concentrations, in a partially TLR2-dependent but TLR4-independent manner [35].

**Figure 4:**
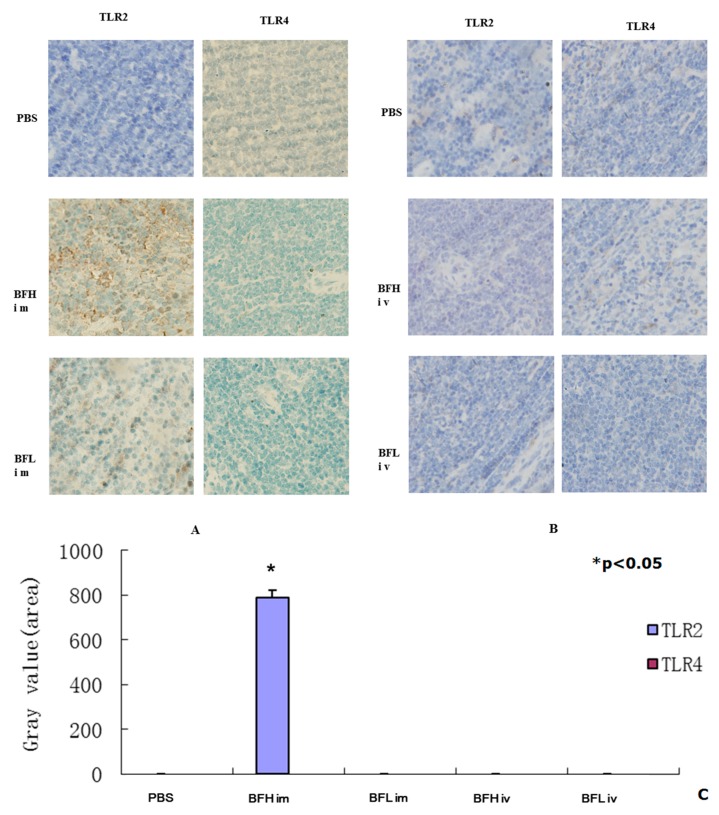
Immunohistochemical (IHC) assay of the spleen of BALB/c mouse via IM and IV treatment with BF-rTK (*n* = 6). (**A**) IM injection of a high dose of BF-rTK induced TLR2 significantly increased expression and TLR4 had no detectable variation; (**B**) TLR2 and TLR4 were undetectable treated with BF-rTK via IV with both high and lower doses of BF-rTKs. The slices were detected three times with duplicate technical replicates; (**C**) Quantitative analysis of TLRs (* *p* < 0.05).

The immunohistochemical (IHC) assay results indicate that TLR2 was significantly upregulated by IM injection of the high dose of BF-rTK (1.0 × 10^6^ cells/mL) and was not upregulated with the low dose treatment (1.0 × 10^4^ cells/mL). In contrast, treatment with the both high and low doses of BF-rTK did not upregulate TLR4 expression by IM injection (Figure 4). However, both TLR2 and TLR4 were not detectable following IV administration of BF-rTK (Figure 4). Thus, these results indicated that IM injection of the high dose of BF-rTK stimulated the immune response through TLR2. However, IV administration of BF-rTK did not activate TLR2. Therefore, IV administration of BF-rTK is superior for tumor gene therapy.

### 2.4. BF-rTK/GCV Treatment Significantly Inhibits Tumor Growth

**Figure 5:**
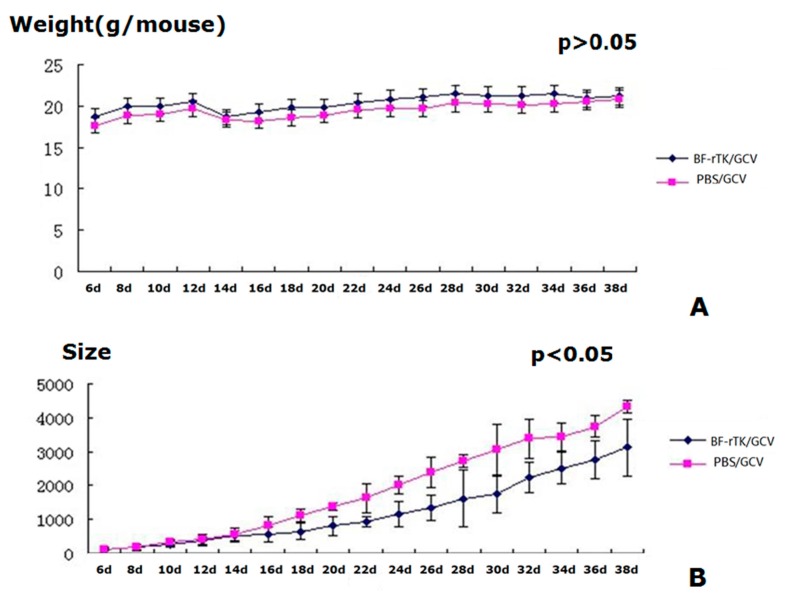
BF-rTK/GCV treatment significantly inhibits tumor growth *in vivo*. (**A**) Body weight curve of nude mice treated by BF-rTK/GCV or PBS/GCV (*p* > 0.05; *n* = 5); (**B**) Tumor growth of nude mice treated by BF-rTK/GCV or PBS/GCV (*p* < 0.05; *n* = 5). The tumor size was calculated as follows: V = 1/2AB^2^ (V: tumor size, A: tumor long diameter, B: tumor short diameter).

BF-rTK/GCV administration had no effect on weight gain in gastric tumor-bearing nude mice compared with the results seen for the PBS/GCV control cohort (Figure 5A). Furthermore, there were no deaths during the experimental period. Tumor growth in BF-rTK/GCV-treated mice, however, was greatly lower than that for PBS controls at 16 days post-administration (*p* < 0.05; Figure 5B). The growth curve of the mice was used as an index to reflect the safety of BF-rTK administration. Therefore, the results indicated that IV BF-rTK/GCV administration was efficient and safe for solid tumor gene therapy.

### 2.5. BF-rTK/GCV Significantly Upregulates Apoptosis-Associated Molecules Expression

**Figure 6:**
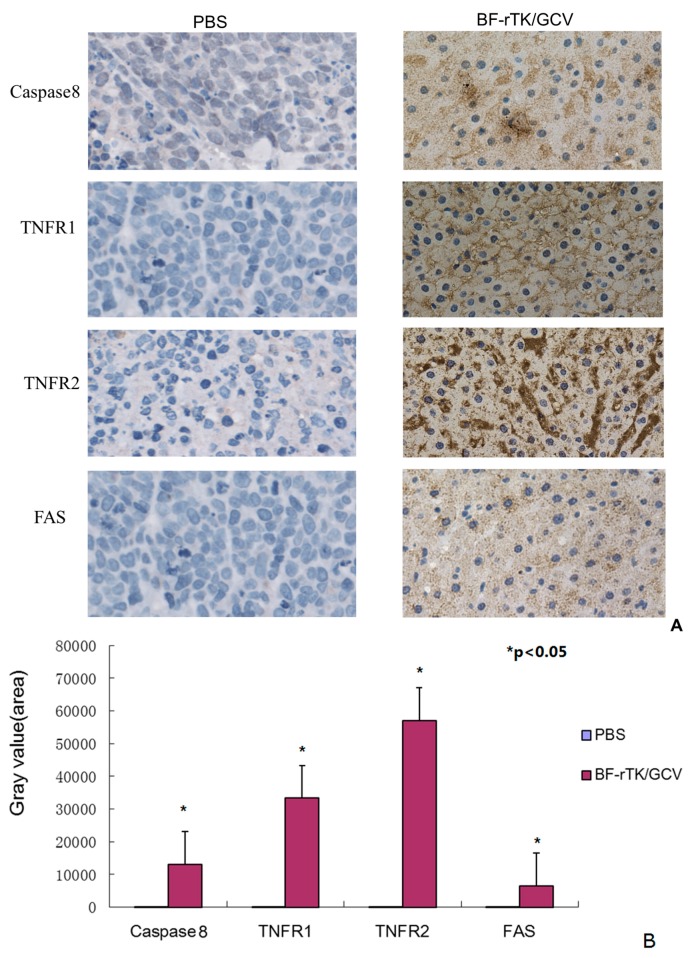
IHC assay of the tumor sample from nude mice treated by BF-rTK/GCV via IV (*n* = 6). (**A**) The tumor tissue samples were from MKN-45 gastric cancer cells treated by PBS/GCV, and BF-rTK/GCV, respectively. The FAS, TNFR1, TNFR2, and caspase 8 levels were analyzed with IHC; (**B**) Quantitative analysis of FAS, TNFR1, TNFR2, and caspase 8 (* *p* < 0.05).

In order to evaluate the antitumor activity and mechanism of *Bifidobacterium* as a gene transfer vehicle, we analyzed the gastric xenograft tumor tissues treated by BF-rTK/GCV against PBS/GCV with apoptosis-associated molecule antibodies. The significantly upregulated expression of caspase 8 indicated that BF-rTK/GCV induced vigorous apoptosis. The upregulated expression of numerous death receptors (TNFR1, TNFR2, and FAS) suggested that BF-rTK/GCV induced tumor cell apoptosis by death receptors signaling pathway in gastric cancer. TNFR2 was the most significantly upregulated molecule in BF-rTK/GCV treatment (Figure 6, *p* < 0.05). However, Fas (CD95) was slightly upregulated compared with TNFRs (Figure 5, *p* < 0.05). The results suggest that BF-rTK/GCV triggered several death receptors mediated signal transduction pathways (e.g., Fas, TNFR1, and TNFR2) and the signals were transduced through the caspase 8 associated pathway.

### 2.6. BF-rTK Regulates Caspase 8 Signaling Apoptosis-Associated Downstream Molecules in Intestinal Cancer

**Figure 7:**
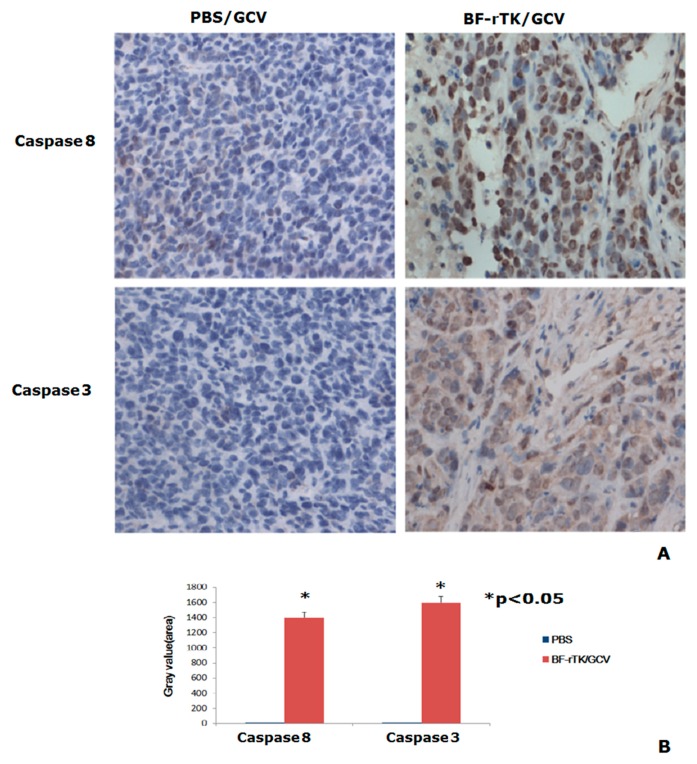
IHC assay of the intestinal cancer colo320 xenograft sample from nude mice treated by BF-rTK/GCV (*n* = 3). (**A**) The tumor tissue samples were from intestinal cancer colo320 cells treated by PBS/GCV, and BF-rTK/GCV, respectively. The caspase 8 and caspase 3 levels were analyzed with IHC; (**B**) Quantitative analysis of caspase 8 and caspase 3 (* *p* < 0.05).

To investigate the universality of caspase 8 signaling apoptosis in human tumor, we tested the caspase 8 and caspase 3 in intestinal cancer colo320 cell line xenografts (*n* = 3) with IHC firstly. The results indicated that the BF-rTK/GCV treatment significantly increased caspase 8 and caspase 3 in intestinal cancer colo320 xenograft tissues comparing with PBS/GCV group (Figure 7). The caspase 8 variation trend was consistent with its expression in gastric cancer model (Figure 6). That meant that the caspase 8 signaling apoptosis in human tumors was universal.

**Figure 8:**
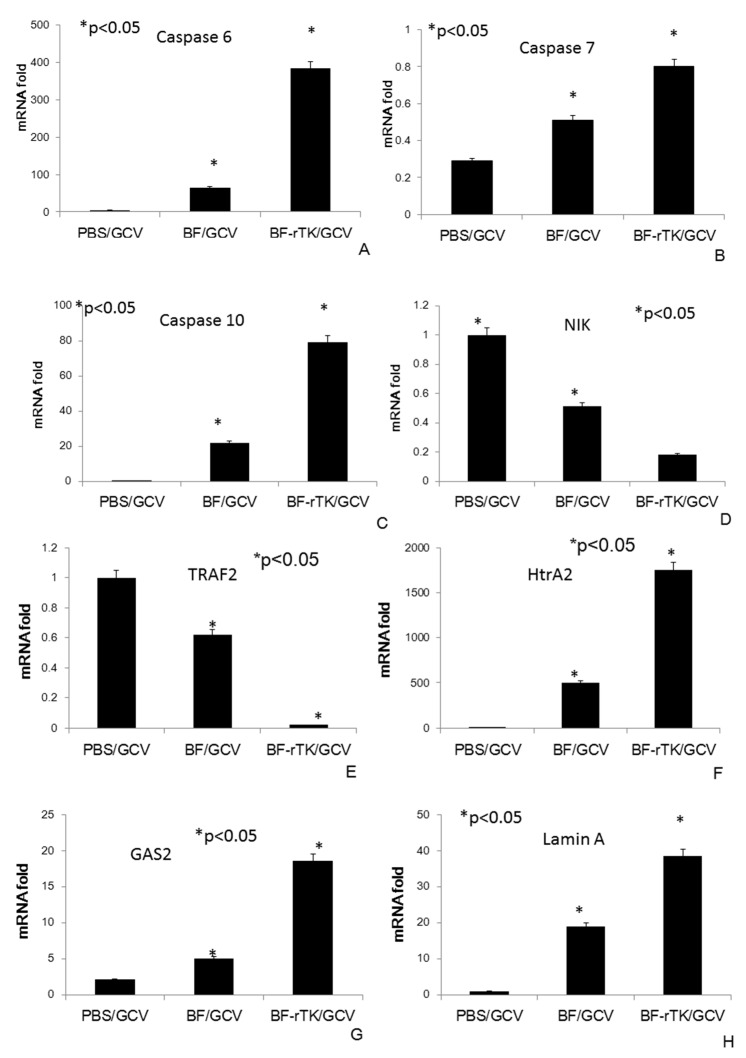
Caspase 8-related apoptosis genes are enriched in rTK gene induced apoptosis. (**A**–**H**) Xenograft tumor tissues treated by PBS/GCV, BF/GCV and BF-rTK/GCV were collected and RNA was extracted from tumor tissues (*n* = 3). Gene expression of caspase 6 (**A**), caspase 7 (**B**), caspase 10 (**C**), NIK (**D**), TRAF2 (**E**), HTRA2 (**F**), GAS2 (**G**), and Lamin A (**H**) were analyzed by qRT-PCR using specific primers. All values were normalized to glyceraldehyde-3-phosphate dehydrogenase (GAPDH) as an internal control and were expressed relative to tumors treated with GCV in each case (* *p* < 0.05).

Therefore, the quantitative real time PCR (qPCR) was used to quantify mRNA abundance of downstream genes of caspase 8 in intestinal cancer colo320 cell line xenografts (*n* = 3). We found that caspase 6, caspase 7, and caspase 10 expression were greatly enriched (Figure 8A) in the group treated BF-rTK/GCV as compared with BF/GCV treatment alone. They were all the caspase 8 activated downstream molecules associated with cancer apoptosis (Figure 8A–C). By contrast, NIK and TRAF2 expression, which were associated with anti-apoptosis activation, were decreased after BF-rTK/GCV treatment (Figure 8D,E). X-linked inhibitor of apoptosis protein (XIAP) was also known as inhibitor of apoptosis protein 3 (IAP3) which inhibited apoptotic cell death. When XIAP was inhibited by HtrA2 (serine protease), it would induce apoptotic cell death [36]. We found that HtrA2 increased significantly after BF-rTK/GCV treatment (Figure 8F). Transcript encoding proteins, Gas2, that functioned downstream of caspase 3 also all increased expression after BF-rTK/GCV treatment (Figure 8G). We detected that Lamin A, which functions downstream of caspase 6, was significantly upregulated in the BF-rTK/GCV treatment group in intestinal cancer (Figure 8H). The results implied that the tumor apoptosis was induced by the upregulated expression of HtrA2, caspase 6, caspase 7, caspase 10, Gas2, and Lamin A.

## 3. Discussion

Cancer has been one of the major global causes of morbidity and mortality in recent decades [37]. Gastrointestinal tumor (gastric cancer, intestinal cancer) is one of the most common cancers worldwide [38]. Although an increasing number of patients can be treated successfully with surgery, interstitial chemotherapy, radiotherapy, and immunotherapy [2], significant efforts have gone into the development of non-traumatic, non-surgical tumor gene therapy technology [2,3,10,13,25,39]. However, the lack of a safe and suitable tumor targeting gene delivery vehicles is still a challenge [2,4,6,8,10,11].

BF has been constructed as a solid tumor gene targeting therapy system with optimal antitumor effects when combined with GCV [9,10,21,22,23,27]. The targeting and colonization of BF in the tumor center were visualized by Cronin *et al.* [40]. However, it is still unclear whether BF is safe as a gene delivery system for solid tumor gene targeting therapy. Here, we demonstrate that IV administration of low-dose BF-rTK is safe in our model. It is reasonable to expect that most of the BF-rTK is eliminated by lymphocytes to obtain a higher partial pressure of oxygen in the circulatory system or in normal tissues. Additionally, BF is a gram-positive bacterium that lacks endotoxins and thus does not evoke cytokine production or induce a violent immune response. In contrast, the effects of IM administration of BF-rTK would last longer, allowing increased time for interaction with lymphocytes and evoking a stronger immune response. Thus, following IV administration, only a small amount of live BF-rTK is transported into the center of the solid tumor, where there is a hypoxic microenvironment.

The question is raised, however, whether this dose is sufficient to effectively treat the tumor. First, BF is alive outside of the tumor cells and expresses TK, which phosphorylates the non-toxic precursor GCV into a toxic substance, ganciclovir-3-phosphate, to kill cancer cells [10]. Therefore, even if only a small amount of live BF-rTK recombinant entered the solid tumor center, it would quickly proliferate independent of the tumor cells. Second, the BF vector differs from viral vectors (e.g., adenovirus (Adv) vector), which are limited by the physiological state of the cancer cell in that the antitumor effect is dependent on the multiplicity of infection (MOI). It has reported that the MOI of Adv-Rous sarcoma virus (RSV)-TK is not less than 66 in MDAH-2774 ovarian cancer cells after acyclovir treatment [11]. That meant that if a cancer cell was simultaneously infected or viral productions were less than 66 viruses, the cancer cell would not be killed by this Adv-RSV-TK treatment. Compared with viral vectors, the superiority of the BF vector was that the bacterium survived intercellular of tumor tissue. Therefore, the recombinant TK gene expression was independent of the intracellular environment of tumor. Thus, BF could deliver sufficient suicide genes into the target tumor tissue without the MOI limitation. The solid tumor targeting feature of BF is depended on the anaerobic microenvironment. Therefore, the application of BF-rTK/GCV is suitable for any kind of solid tumor without receptors or the histological origin and subtype classification of cancer.

Immunological injury induced by gene therapy vectors is a major cause of patient death [7,41]. Therefore, the immune and/or inflammatory reaction is a critical index for evaluating the safety of this gene therapy vector. Cytokines play important roles in the immune and/or inflammatory responses of vectors in cancer gene therapy. It was reported that the induction of cytokines by gene therapy vectors was a side effect in patients and had a negative effect on the viral vectors. For example, the AdV vector stimulated IL-12 release, which, in BALB/c mice, correlated with increased AdV vector clearance [33]. The high levels of IFN-α produced were critical for both the innate and adaptive immune responses against AdV vectors [16]. The increased concentrations of IL-6, IL-8, and TNF-α were associated with febrile peaks, septic shock, and the severity of AdV infection in children [34,42]. However, our study indicated that systematic administration of BF-rTK did not evoke such kinds of cytokine expression. This finding is similar to previous reports indicating that BF decreases pro-inflammatory cytokines in human dendritic cells [43,44]. However, there was no evidence that cytokines inhibited BF survival in tumor tissues.

Tumor angiogenesis is thought to be fundamental in tumor development and progression. Vascular endothelial growth factor (VEGF) has been used for evaluation of neoangiogenesis in tumors [45,46,47]. It is concerned about whether BF-rTK/GCV induces neoangiogenesis of tumor after IV or IM administration. This study showed that BF-rTK/GCV administration (IV or IM) significantly downregulated VEGF expression (Figure 2A,C). The result suggested that besides inducing tumor apoptosis, BF-rTK/GCV administration (IV or IM) also inhibited the tumor angiogenesis. It is a good topic to explore the mechanism of BF-rTK/GCV inducing anti-tumor angiogenesis in the future.

As a summary, the safety evaluation included serum profiling of inflammatory cytokine following IV and IM administration of BF-rTK/GCV and its comparison to PBS/GCV cohort. IM administration (at high and low doses) resulted in upregulation of several inflammatory cytokines, suggesting that the tested doses and the routes may not be preferable. However, IV administration, particularly at low doses, did not result in upregulation of the majority of the inflammatory cytokines significantly more than that of the control group, suggesting this dose and route might prove to be a safer option.

## 4. Experimental Section

### 4.1. Construction of the BF-rTK Gene Therapy System

The HSV TK gene (accession AB032875) was PCR amplified and cloned into pBEX at the *Bam*H I and *Sal* I sites. pBEX was constructed by Ma *et al.* [48] and used as the expression vector for BF. Potential recombinants were first screened by PCR. The *tk* sense primer was GATATCTACGGATCCATGGCTTCGTACCCCTG and the antisense primer was GTATCAGGTCGACGTTAGCCTCCCCCATC. 6 × His tag was added in the N-terminus of TK by PCR. PCR was performed under the following conditions: 1 cycle of 95 °C for 2 min; 25 cycles of 95 °C for 30 s, 55 °C for 20 s, and 72 °C for 2 min; and 1 cycle of 72 °C for 5 min. The recombinant plasmid was transformed into competent *Bifidobacterium infantis* via electroporation and verified by PCR and PAGE analyses. The TK was purified by magnetic Nickel beads (Genscript, Nanjing, China) as the protocol described. Briefly, (1) His-TK was affinity absorbed with 1 mL Ni-IDA resin (Genscript, Nanjing, China) at 4 °C overnight; (2) Then the Ni-His-TK-IDA resin was washed three times with PBS. The resins were washed with washing buffer (0.1% Triton in 1 × PBS) three times to remove the nonspecific interacting proteins; (3) The TK was eluted with elution buffer (imidazole buffer, 50–200 mM) and the samples were desalted with semipermeable membrane at 4 °C overnight following the final washing; (4) Then the sample was enriched with PEG6000 at 4 °C overnight for SDS PAGE assay.

BF or BF-rTK (pBEX-*tk*) cells (0.5 mL, 2.0 × 10^5^ cells/mL) were prepared and mixed with 1.0 mL GCV (5.0 mg/kg). The negative control was 0.5 mL PBS mixed with 1.0 mL GCV (5.0 mg/kg) and using PBS for short in the text.

### 4.2. Experimental Animals

This study was carried out in strict accordance with the recommendations in the Guide for the Care and Use of Laboratory Animals of the National Institutes of Health. The protocol was approved by the Committee on the Ethics of Animal Experiments at the Chongqing Medical University (SYXK2012-0001). All surgery was performed under sodium pentobarbital anesthesia, and all efforts were made to minimize suffering.

BALB/c and BALB/c nude mice (male, 3–4 week, 18–20 g/mouse) were housed at the Laboratory Animal Center of Chongqing Medical University (Chongqing, China). Animals were housed in microisolator cages under specific pathogen-free conditions with 12 h light-dark cycles for 38 days. Animals were weighed at regular intervals and monitored daily.

A humane endpoint was used for the early euthanasia of animals; humane endpoints were judged by observing animals for physiological signs of hunched posture and lethargy. Animals were anesthetized with sodium pentobarbital and sacrificed by cervical dislocation.

### 4.3. Cells and Cell Culture

The MKN-45 gastric cancer cell line was obtained from the Committee on Type Culture Collection of Chinese Academy of Sciences and maintained under the following conditions: RPMI 1640 medium with 2 mM l-glutamine, 1.5 g/L sodium bicarbonate, 4.5 g/L glucose, 10 mM HEPES, 1.0 mM sodium pyruvate, and 10% fetal bovine serum. The cells were cultured in 100 mm culture dishes in a humidified, mixed environment of 37 °C and 5% CO_2_.

### 4.4. Evaluation of the Safety of the BF-rTK/GCV System by a Cytokine Profile Assay

A preliminary trial investigating IV gene therapy in nude mice indicated that 1.0 × 10^6^ cells/mL of BF-rTK was the highest concentration with no adverse effects, whereas 1.0 × 10^4^ cells/mL was the lowest effective concentration. At concentrations greater than 1.0 × 10^7^ cells/mL, IV injection resulted in venous embolisms and subsequent death. Based on these results, this study tested the effect of high (1.0 × 10^6^ cells/mL) and low (1.0 × 10^4^ cells/mL) doses of BF-rTK.

Thirty BALB/c mice were divided randomly into four groups. Group A was IV administered 200 μL of the low dose (1.0 × 10^4^ cells/mL) of BF-rTK (BFL IV; *n* = 6), whereas group B was administered 200 μL of the low dose (1.0 × 10^4^ cells/mL) via IM injection (BFL IM; *n* = 6). Group C received an IV injection of 200 μL of the high dose (1.0 × 10^6^ cells/mL) of BF-rTK (BFH IV; *n* = 6), whereas group D received an IM injection of the high dose (1.0 × 10^6^ cells/mL; BFH IM; *n* = 6). As a negative control, PBS (200 μL) was injected IV (*n* = 3) or IM (*n* = 3). Another four groups of BALB/c mice were administered *Escherichia coli* DH5α under the same conditions as BF-rTK treatment. Blood was sampled 24 h post-injection and serum was isolated for cytokine profile analysis. The spleen was collected from BF-rTK-treated mice and fixed in a 10% formaldehyde solution for IHC analysis.

The serum cytokine profile was analyzed with a mouse cytokine antibody array (cat# QAM-CYT-1, RayBiotech, Norcross, GA, USA) after BF-rTK or *E. coli* DH5α treatment. Briefly, antibody arrays were blocked with blocking buffer for 30 min; and 100 μL aliquots of cytokine standards or BF-rTK/GCV-treated samples were added into each well. Samples were incubated for 2 h at 4 °C, and the plate was then washed three times. A cocktail of biotin-conjugated antibody was added, and samples were incubated for 2 h at 4 °C; then, the plate was then washed three times. Cy3 equivalent dye-labeled streptavidin was added and samples were incubated for 1 h in the dark at room temperature. Signals were detected and the data were analyzed using the RayBiotech cytokine antibody array software (RayBiotech, Code: QAM-CYT-1, Norcross, GA, USA, 2015) tool. A value of *p* < 0.01 was considered significant.

### 4.5. Immunohistochemical (IHC) Detection of Toll-Like Receptors Upregulated by BF-rTK/GCV Treatment

The spleen was collected from BF-rTK-treated mice, embedded in paraffin, and serially sectioned (4 μm) for analysis. Sections were deparaffinized, rehydrated, and subjected to antigen retrieval followed by 3% hydrogen peroxide treatment. The following primary antibodies (1:100) were added: rabbit polyclonal anti-TLR2 (BA1716) and anti-TLR4 (BA1717) (BOSTER, Wuhan, China). Samples treated with primary antibodies were incubated overnight at 4 °C. They were then treated with biotinylated goat anti-rabbit antibody and incubated for 30 min at 37 °C followed by incubating with horseradish peroxidase-labeled streptavidin for 15 min at 37 °C. The sections were stained, dehydrated, made transparent, and subjected to blocking for analysis.

### 4.6. Establishment of Xenograft Tumor Models and Assessment of the Efficacy of Inhibition Tumor Growth by BF-rTK/GCV Treatment

A xenograft tumor mouse model was established by subcutaneous injection of MKN-45 gastric cancer cells (1.0 × 10^8^ cells/mL) in two separate areas of the body. Ten tumor-bearing nude mice were randomly divided into two groups, PBS + GCV (PBS/GCV, *n* = 5; control) or BF-rTK/GCV (*n* = 5), at 13–14 days after the xenograft. In order to obtain the best effect of inhibiting tumor, we used only the higher dose in survival curve test. Each group was administered a single dose of PBS/GCV or BF-rTK/GCV through the tail vein injection (BF or BF-rTK: 1.0 × 10^6^ cells/mouse; GCV: 5.0 mg/kg). The mice were then administered GCV (5.0 mg/kg) daily by intraperitoneal injection. Cancer growth (size of the tumor) and the number of surviving mice were measured after PBS/GCV or BF-rTK/GCV treatment. The tumor size was calculated as follows: V = 1/2AB^2^ (V: tumor size, A: tumor long diameter, B: tumor short diameter).

### 4.7. IHC Detection of Apoptosis-Associated Molecular Markers Activated by BF-rTK/GCV Treatment

IHC of active caspase 8 (apoptosis-related cysteine peptidase) and death receptors (TNFR1, TNFR2 and FAS) were performed on a gastric tumor tissues treated by PBS/GCV and BF-rTK/GCV, respectively. Retrieved tissues were fixed, decalcified in 10% formalin and embedded in paraffin 24 h posttreatment. Serial sections of the embedded specimens were stained with hematoxylin and eosin (H & E). The fixed tissues of colo320 intestinal tumor were blocked and incubated with caspase 8 (P18) antibody (BA2143-2, Boster, Wuhan, China), and caspase 3 (P12) antibody (BA3592, Boster), The fixed tissues of MKN-45 gastric cancer tumor colo320 intestinal tumor were blocked and incubated with caspase 8 (P18) antibody (BA2143-2, Boster), TNFR1 antibody (BA4891), TNFR2 antibody (BA1438) and FAS antibody (BA0048-1). After being washed, tissues were incubated with biotin-labeled secondary antibody for 30 min, followed by incubating cells with streptavidin-HRP conjugate for 20 min at room temperature (RT). The presence of the expected protein was visualized by diaminobenzidine (DAB) staining and examined under a microscope. Stains with control IgG were used as negative controls.

### 4.8. RNA Isolation and Quantitative RT-PCR

In order to elucidate the university of BF-rTK/GCV anti-tumor effect was associated to the caspase 8 signaling pathway, another group of intestinal cancer colo320 model was established as previously described (*n* = 6). Total RNA was extracted from three intestinal cancer colo320 tissues treated by PBS/GCV, BF/GCV and BF-rTK/GCV after 24 h, respectively, using TRIzol reagent (Invitrogen, Waltham, MA, USA). Following ethanol precipitation, total RNA was applied to an RNase column (Qiagen, Venlo, The Netherlands) for further purification and treated with DNase following the manufacturer’s protocol. The cDNA was synthesized from 1 μg total RNA using the SuperScript III reverse transcriptase kit (Invitrogen) in a final volume of 20 μL. Primers were designed with the IDT SCI primer design tool (Integrated DNA Technologies, San Diego, CA, USA). Quantitative real-time PCR experiments were performed with Bio-Rad MJ MiniOption Real-Time PCR System in triplicate and the data were analysis by CFX manager software version 1.5 (Bio-Rad Laboratories, Inc., Berkeley, CA, USA). The PCR data were normalized to GAPDH expression. Sequence and product lengths for each primer pair were listed in Table S1.

### 4.9. Statistical Analysis

The data were analyzed using the SPSS19.0 software (IBM, Armonk, NY, USA). Statistical comparison between two groups was performed using the Student’s *t*-test. For comparison of three or more groups, we used one-way analysis of variance. *p* < 0.05 was considered statistically significant.

## Supplementary Materials

Supplementary materials can be found at http://www.mdpi.com/1422-0067/17/6/891/s1.
