# Supplementary material for: Intravenous Administration Is an Effective and Safe Route for Cancer Gene Therapy Using the Bifidobacterium-Mediated Recombinant HSV-1 Thymidine Kinase and Ganciclovir
Source: Int J Mol Sci. 2016 Jun 6;17(6):891. doi: 10.3390/ijms17060891 (PMC4926425; doi:10.3390/ijms17060891)
Supplement: Supplementary file 1 [file ijms-17-00891-s001.pdf]

Supplementary Materials: Intravenous Administration Is an Effective and Safe Route for Cancer Gene Therapy Using the *Bifidobacterium*-Mediated Recombinant HSV-1 Thymidine Kinase and Ganciclovir

Huicong Zhou, Zhiliang He, Changdong Wang, Tingting Xie, Lin Liu, Chuanyang Liu, Fangzhou Song and Yongping Ma

Table S1. Primers used in this study.

Primers	Sequence
GAPDH sense	5′-ACCACAGTCCATGCCATCAC-3′
antisense	5′-TCCACCACCCTGTTGCTGTA-3′
Caspase 6 sense	5′-GCCGATTGCTTTGTGTGTGT-3′
Caspase6 antisense	5′-ACCATGAGCCGTTCACAGTT-3′
Caspase 7 sense	5′-GGGCCCATCAATGACACAGA-3′
Caspase 7 antisense	5′-GTCTTTTCCGTGCTCCTCCA-3′
Caspase 10 sense	5′-ACCAAAGAGGAAGTGGAGCG-3′
Caspase 10 antisense	5′-CTGCGGTAAGGCTTCCAAGA-3′
Gas2 sense	5′-GCCCCTTCTCCTTCACCTTC-3′
Gas2 antisense	5′-CCTGCAAAAGTTTCCCAGCC-3′
HtrA2 sense	5′-AGGGGAGTTTGTTGTTGCCA-3′
HtrA2 antisense	5′-GGTCAGCATCATCACCCCAA-3′
Lamin A sense	5′-CATGGAGATCCACGCCTACC-3′
Lamin A antisense	5′-GGGAACCGGTAAGTCAGCAA-3′
NIK sense	5′-CAGAAGGAACTCCCCAAGCCAAA-3′
NIK antisense	5′-CGACGCTTTCCCTTCCAACA-3′
